# Understanding the Overlap: Exploring the Complex Comorbidity of ASD and ADHD

**DOI:** 10.1192/j.eurpsy.2025.1172

**Published:** 2025-08-26

**Authors:** A. Rodríguez-Quiroga, A. Álvarez Astorga, A. M. Matas Ochoa, J. Quintero

**Affiliations:** 1 Psychiatry, Hospital Universitario Infanta Leonor, Madrid, Spain

## Abstract

**Introduction:**

Autism Spectrum Disorder (ASD) and Attention-Deficit/Hyperactivity Disorder (ADHD) are neurodevelopmental conditions that often co-occur, leading to complex clinical presentations. While ASD is characterized by deficits in social communication and restricted, repetitive behaviors, ADHD is marked by inattention, hyperactivity, and impulsivity. The comorbidity between these conditions is increasingly recognized, yet their combined impact on diagnosis, treatment, and patient outcomes remains underexplored.

**Objectives:**

This study aims to investigate the prevalence and nature of the comorbidity between ASD and ADHD. We also seek to identify the shared and distinct cognitive, behavioral, and developmental features, and assess the implications of this overlap for clinical practice, especially in diagnosis and treatment planning.

**Methods:**

A systematic literature review was conducted, examining peer-reviewed studies published in the last 10 years. Key databases such as PubMed, PsycINFO, and Scopus were searched for studies involving ASD and ADHD comorbidity in children and adolescents. Data on prevalence rates, diagnostic criteria, symptom overlap, and treatment approaches were extracted and analyzed.

**Results:**

The findings confirm a high prevalence of comorbidity between ASD and ADHD, with estimates ranging from 30% to 50% in pediatric populations. Shared symptoms, particularly inattention and executive dysfunction, often complicate differential diagnosis. Children with both ASD and ADHD tend to exhibit more severe social and cognitive impairments, and have a higher risk for anxiety, mood disorders, and academic challenges. The results suggest that overlapping symptoms may delay or complicate accurate diagnosis, affecting treatment efficacy.

**Conclusions:**

The comorbidity of ASD and ADHD presents unique challenges for clinicians and families. Early identification of both conditions is crucial for tailored interventions. A multidisciplinary approach, combining behavioral, cognitive, and pharmacological treatments, appears to be the most effective. Further research is needed to develop clearer diagnostic criteria and targeted therapeutic strategies to address the specific needs of individuals with this comorbid profile.

**Disclosure of Interest:**

None Declared

